# Conceptualizing the studies of time perception in spinal cord injury

**DOI:** 10.3389/fnint.2026.1711553

**Published:** 2026-02-13

**Authors:** Ekaterina D. Slovenko, Pavel E. Musienko, Olga V. Sysoeva

**Affiliations:** 1 Laboratory of Human Higher Nervous Activity, Institute of Higher Nervous Activity and Neurophysiology, The Russian Academy of Science, Moscow, Russia; 2 Laboratory of Neurostimulation Technologies, Federal Center of Brain Research and Neurotechnologies, Moscow, Russia; 3 Department of Neuroscience, Sirius University of Science and Technology, Bolshoy Sochi, Krasnodar Krai, Russia; 4 Institute of Translational Biomedicine, Saint Petersburg State University, Saint Petersburg, Russia; 5 Moscow Center for Advanced Studies, Moscow, Russia; 6 Center for Cognitive Science, Sirius University of Science and Technology, Bolshoy Sochi, Krasnodar Krai, Russia; 7 Faculty of Biology and Biotechnology, HSE University, Moscow, Russia

**Keywords:** embodied cognition, interoception, sensorimotor synchronization, spinal cord injury, time perception

## Introduction

Sense of time is important for comprehension of both the outer environmental events and the inner corporeal processes. Research of time perception under different pathological conditions allows to reveal features of this process that may go unnoticed in healthy individuals. Knowledge of injury-related perceptual bias can be used for diagnostic and therapy of cognitive decline following physical limitations. The role of the spinal cord in time perception and other cognitive functions is not apparent, as its primary functions are consciously uncontrolled visceral and locomotor automatisms, conduction of the sensorimotor signals. However, current models of timing consider sensory information and motion as important factors that shape the temporal sense (Basgol et al., 2020; De Kock et al., 2021). In this paper we take a brief view of theoretical and empirical findings indicative of possible changes of time perception due to spinal cord injuries (SCI). In the discussion we present our perspective of questions worth attention in future research.

## Spinal neural pathways involved in timing system

Time perception includes the complex process of aligning external events with the previously acquired mental representations, which requires sensory input through different modalities and engages both central and peripheral parts of the nervous system. Spinal cord relays the sensory and motor signals from peripheral neurons to the brain and regulates the autonomous visceral and locomotor activity. These functions are essential for time perception, which is inextricably connected to sensation and motion.

## Sensation and time perception

As the brain is the highest level of cognitive processing, where all the sensor and motor processes converge, most studies of the time-keeping system are concerned with cortical and subcortical structures. Empirical findings confirm the differentiated contribution of modality-specific and associative cortices in time perception (Bueti, 2011). While exteroception is involved in assessment of external events duration, interoception is presumed to form the reference time measures, and the integrative role is attributed to the insular cortex (Craig, 2009). The insular cortex was proved to be engaged in explicit timing tasks (Wittmann et al., 2010), as well as emotionally charged bodily sensations (Craig, 2002). Time perception has also shown to be influenced by pain, which indicates the links between the feeling of time and the protopathic sensitivity (Ogden et al., 2015; Rey et al., 2017). Importantly, the concept of interoception shall be distinguished from visceroception as it includes all body feelings irrespective of their internal or external location, as well as their emotional and cognitive interpretation (Berntson and Khalsa, 2021). While the substantial part of visceral sensations are carried by the vagal nerve, the temperature, pain, itch, and touch sensations are transferred through the dorsal horn, among all the sensation from the limbs and the trunk (Wang et al., 2022). Thus, if the temporal sense is based on interoceptive signals, distortion of temperature, pain and tactile sensations after a SCI presumably shall significantly influence the time perception.

## Motion and time perception

The interconnection of time perception and motor system are also widely studied on the brain level: the involvement of the supplementary motor area (SMA) in explicit timing tasks was confirmed using neuroimaging (De Kock et al., 2021) as well as basal ganglia and cerebellum (Fontes et al., 2016)—the areas primarily engaged in motion and postural control. Time perception distortions are also present in Parkinson's disease—a neurodegenerative disorder accompanied by movement impairment due to basal ganglia disruption (Honma and Terao, 2024). The functions of the motor areas in time perception may be developed during life through active sensing, which consists in the motor learning of temporal relations between actions and their consequences. Empirical studies show that movements can either bias time perception or improve it depending on the motion's direction, rhythmicity, effector, vigor and the possibility for synchronization (De Kock et al., 2021).

Locomotion shall have a special impact on timing as a whole-body motion often requiring combination with temporal judging, because moving often requires taking account of spatio-temporal deployment of external events. However, only particular aspects of interaction between locomotion and timing were revealed in studies in healthy participants under treadmill conditions: irregular or increased locomotion pace decreases the accuracy and the certainty of time estimations, indicating weakened temporal control during strenuous walking (Castellotti et al., 2022; Kroger-Costa et al., 2013). Since simultaneous execution of cognitive non-temporal tasks enhanced the effect (Castellotti et al., 2022), these results are consistent with the attention allocation model, suggesting a competition between cognitive tasks for the limited attention resource. However, the relation between self-paced walking in field conditions and time perception is yet to be explored.

This evidence suggests that the sense of time and motion are interdependent in a fully developed neural system unconstrained by neurological deficits. However, the nature of this connection is not fully understood, which would be important for modeling the time perception mechanisms.

## Time perception in spinal cord injury

The disruption of spinal neural pathways sets specific conditions for brain functioning: the sensory input from inner organs and external receptors are selectively restricted along with the sensorimotor feedback loops. In chronic injury the brain is gradually reorganized in accordance with the changes of the sensory and motor pathways: there is evidence of changes in the somatotopy of the sensory area (Jurkiewicz et al., 2007) and the morphological properties of neurons in sensory and motor areas (Wrigley et al., 2009). Given the anatomical changes of the somatosensory cortex, changes of body-grounded cognitive functions are expected.

As opposed to the view of cognition as a symbolic system independent of the sensorimotor system, embodied cognition theories state that all human experience is grounded in the body (Moro et al., 2022). The principal difference of these two approaches is the view of the body's role in connection between the objective world and cognition. Cognitive changes associated with sensory or motor deprivation may explain the contribution of the bodily experience in development of higher-order mental processes. Spinal cord injury is considered as a unique situation of body-brain disconnection with spared cognitive functions but limited sensory input. There is empirical evidence of decrease in temporal assessment accuracy associated with reduced interoceptive sensitivity in patients with SCI (De Martino et al., 2022). Importantly, the degree of temporal and interoceptive sensitivity reduction was proportional to the injury level, which points out the foundation of the temporal sense on a range of distributed bodily signals. The personal meaning of time in patients with SCI was studied through analysis of narratives (Seymour, 2002; Sparkes and Smith, 2003). In this approach not the accuracy and precision of interval timing is considered, but the tempo, the circularity and the continuance of long-lasting life events. It was found that the role of time changes drastically after the injury, when the habitual life tempo is replaced by time-bound rehabilitation interventions and the recovery timelines become the main determinant of the re-entry to ordinary life. Strict timekeeping prescribed by the treatment regime becomes essential for survival and well-being. These insights emphasize the importance of incorporating time perception function assessment for people undergoing SCI and rehabilitation.

## Discussion and research perspective

Theoretical approaches discussed above indicate that sensorimotor functions affected by spinal cord damage have influence on time perception and particular effects were implicitly confirmed by empirical studies in able-bodied participants. Direct evidence could be obtained from experimental studies of people with spinal cord injuries. The possibility to find homogenous changes is questionable given the difference of post-injury consequences and pre-injury conditions of the patients. But some effects of trauma can be distinguished if consideration is taken of participants' individual characteristics and methodological issues of time perception research.

As regards the individual differences, it is important to assess the quantity and the quality of the spared neural functions, which depend on the level and the extent of the lesion. This factor determines how much sensory information is available for the brain and how wide is the available motion range. The duration of injury shall be taken into account, because the period of active remapping is limited to a few months after injury. So, patients in the acute period of injury can experience specific changes in time perception, while chronic patients can have temporal perception adapted to the SCI deficits and probably equally accurate as in able-bodied people, but with qualitative specificity due to compensatory mechanisms.

Further, comorbid neurological and psychological conditions indirectly related to the spinal injury were shown to affect time perception (Teixeira et al., 2013). In particular, depression is frequently reported after injury (Williams and Murray, 2015), which raises the question of the link between serotonergic system, time perception and depression (Medvedeva et al., 2023). The accuracy of time estimation is also determined by the overall cognitive capacity, genetic predisposition, sociodemographic factors (Bartholomew et al., 2015) as well as inherent differences of time flow stemming from the neurotransmitters balance (Sysoeva et al., 2010; Akhmirov et al., 2024; Marinho et al., 2018). A detailed clinical and psychological examination is necessary to control inter-group differences among patients while the causal link between the degree of deficits in the timing system and depression cannot be excluded. The influence of the interventions and training is also worth attention, as the effects of injury on cognitive performance can be increased or decreased depending on the rehabilitation program. Such rehabilitation tools as brain-computer interfaces, virtual reality or special timekeeping exercises can specifically affect the time perception among other mental capacities.

As for research tools, the primary question is the suitability of the experimental paradigm to the studied temporal domain, as different aspects of time perception are captured by distinct tasks. A preliminary sensory and motor examination can be used to reveal the patient's prevailing deficits and thus to substantiate the choice of temporal perception task. To assess the relation between the sensorimotor functions and the time perception, electrophysiological and neuroimaging techniques can be used. According to functional magnetic resonance imaging studies a number of brain areas involved in somatosensory processing and movement performance are active during interval timing, including the cerebellum, the insular cortex, the basal ganglia, the supplementary motor area (SMA) and the posterior parietal cortex (Li et al., 2022). Neuroimaging of interval timing in patients with sensorimotor deficits could clarify whether changes of time perception are mediated by functional alterations in the corresponding brain structures. Electroencephalogram (EEG) enables examination of neurophysiological correlates of cortical networks engaged in particular cognitive functions. Thus, time perception is characterized by changes in cortical oscillations specific for different duration ranges (Wiener and Kanai, 2016; Rogachev and Sysoeva, 2023) and source localization techniques allow to identify the involved structures (Proshina et al., 2024). During interval timing it is possible to record event-related potentials (ERP) with several ERP components being associated with the timing function. The dependence of time perception on the attention during dual-tasks may be studied using mismatch negativity—the ERP component reflecting the pre-attentive change detection (Näätänen et al., 2004). As for selective attention, it was shown to impact both the point of subjective simultaneity and the amplitude of visual ERP in visuotactile temporal order judgement task (Vibell et al., 2007) and the contingent negative variation reflects the activity of the SMA during anticipation (for a review of EEG correlates of interval timing see Merchant and De Lafuente, 2024). If observed in patients with SCI, changes of these neurophysiological patterns may indicate the reorganization of cortical networks induced by cerebral deafferentation and deefferentation. Analysis of brain activity supplements the psychophysical and psychological evaluation and allows to uncover the connection between the perceptual experience and the underlying physiological processes.

## Conclusion

The above considerations aim at exemplifying the general idea to study time perception in spinal cord injury rather than at extensive review of methodology and research frameworks in this area. In our opinion, current neurophysiological and psychological tools could provide valuable insights if applied to examination of people faced with reorganization of the spinal neural pathways. The accumulation of empirical evidence is essential for establishment of appropriate timelines, combinations and assessment criteria of the tools, enabling their integration into clinical practice. Knowledge of possible injury-related temporal misperception could assist the neurologists in diagnostic test selection and providing the necessary psychoeducation to the patients and their caregivers. Thus, patient care could become more comprehensive.
